# Comorbidities, long‐term outcome and poststroke epilepsy associated with ischemic stroke – A multicenter observational study of 125 dogs

**DOI:** 10.1111/jvim.17291

**Published:** 2024-12-23

**Authors:** Cecilia‐Gabriella Danciu, Rita Gonçalves, Carrete Jordina Caldero, Christoforos Posporis, Javier Espinosa, Steven de Decker, Hanne Gredal, Sophie Elizabeth Wyatt

**Affiliations:** ^1^ Clinical Science and Services Royal Veterinary College Hatfield UK; ^2^ Small Animal Teaching Hospital University of Liverpool Liverpool UK; ^3^ Neurology and Neurosurgery Service Pride Veterinary Referrals, IVC Evidensia Derby UK; ^4^ Department of Veterinary Clinical and Animal Science University of Copenhagen Copenhagen Denmark

**Keywords:** cerebrovascular accident, comorbidities, ischemic infarct, prognosis, seizures

## Abstract

**Background:**

Little is known regarding the comorbidities and prognostic factors associated with the long‐term outcome of ischemic stroke in dogs. Although poststroke epilepsy is a well‐recognized syndrome in people, it is unclear if this phenomenon also occurs in dogs.

**Hypothesis/objective:**

Document comorbidities, long‐term outcome (survival and stroke recurrence), and occurrence of epileptic seizures associated with ischemic stroke.

**Animals:**

One hundred and twenty‐five client‐owned dogs.

**Methods:**

Multicenter observational study including dogs diagnosed with ischemic stroke between 2000 and 2021. Associations between comorbidities, stroke location and extent, poststroke epileptic seizures, and long‐term outcome were investigated. Referring veterinarians and owners were contacted to obtain follow‐up information.

**Results:**

Fifty‐two dogs (41.6%) had a comorbidity. The most common comorbidities were hypertension (20%) and proteinuria (8%). Eight dogs (6.4%) that did not survive to discharge had a territorial ischemic stroke. Overall median survival time for dogs with a comorbidity was 482 days (range, 1‐3013) and 907 days (range, 1‐3027) in dogs without comorbidities (Kaplan‐Meier survival analysis *P* = .602). Twenty‐four dogs (19.2%) had a suspected stroke recurrence and a total of 8/109 dogs (7.3%) developed poststroke epilepsy. No association was found between suspected stroke recurrence or development of poststroke epilepsy and survival (*P* = .812, *P* = .487).

**Conclusions and Clinical Importance:**

Despite no significant difference in survival of dogs diagnosed with ischemic stroke, with or without comorbidities, investigations for underlying causes are recommended to provide appropriate treatment. Poststroke epilepsy is uncommon.

**Abbreviations**
CKD: chronic kidney disease
CSF: cerebrospinal fluid
IQR: interquartile range
LDDST: low‐dose dexamethasone suppression test
MRI: magnetic resonance imaging
PLN: protein‐losing nephropathy
QOL: quality of life
T4: total thyroxine
TSH: thyroid‐stimulating hormone
TEG: thromboelastography
VCM: viscoelastic coagulation monitor

## 1 INTRODUCTION

Ischemic stroke is characterized by neurological deficits secondary to focal ischemic injury involving a defined vascular territory of the brain.^1^ With the wider availability of advanced imaging, ischemic stroke is increasingly recognized in dogs.^2^ Comorbidities such as hyperadrenocorticism and chronic kidney disease are documented in approximately 50% of dogs diagnosed with ischemic stroke.^3^ Although the prognosis for dogs diagnosed with ischemic stroke is generally good,^3^, ^4^ dogs with a comorbidity have shorter survival time compared to those without.^3^ Additionally, up to one‐third of the dogs diagnosed with ischemic stroke experience recurrence of signs of neurological disease which worsened case outcome.^3^, ^4^ Outcome of dogs with ischemic stroke is described in the veterinary literature^3^, ^4^, ^5^; however, the study cohorts are small and recent literature is lacking.

Poststroke epilepsy is a well‐recognized phenomenon in human patients.^6^, ^7^ Although it is generally thought of as an uncommon consequence of ischemic stroke in dogs,^3^, ^4^, ^5^, ^8^, ^9^, ^10^ previous case series have documented epileptic seizures, or epilepsy following diagnosis of ischemic stroke up to 20% in dogs.^3^, ^4^, ^5^ Furthermore, several studies documented epileptic seizures as part of the acute presentation of ischemic stroke in up to 59% of dogs.^4^, ^5^, ^9^ A direct link between epileptic seizures, poststroke epilepsy, and survival has not been established.^4^, ^5^

Given the limited information concerning the long‐term outcome of dogs diagnosed with ischemic stroke, further investigation regarding prognostic factors including comorbidities is needed. Additionally, veterinary literature specifically evaluating the occurrence of poststroke epileptic seizures and epilepsy alongside survival in dogs is currently limited.

The aims of this study were therefore to document comorbidities, stroke location and extent, and association between these variables and long‐term outcome including survival, stroke recurrence, and development of poststroke epileptic seizures in dogs diagnosed with ischemic stroke.

## 2 MATERIALS AND METHODS

### 2.1 Study design

This observational multicenter study, which included 4 veterinary referral hospitals, was approved by the Social Science Research Ethical Review Board of the Royal Veterinary College (SR2022‐0086 and SR2022‐0087). Records of dogs diagnosed with ischemic stroke between January 2000 and December 2021 were retrospectively reviewed.

All dogs were client‐owned and assessed by a board‐certified veterinary neurologist or a neurology resident‐in‐training under the direct supervision of a board‐certified veterinary neurologist. Dogs were included if they had an acute (1‐7 days) or peracute (<24 hours) onset of clinical signs,^11^ which were nonprogressive beyond 24 hours, compatible with an intracranial neuroanatomic localization, and providing magnetic resonance imaging (MRI) findings were consistent with previously published criteria for canine ischemic stroke.^8^, ^9^, ^10^ All included cases required hematology, serum biochemistry, and full urinalysis as a minimum. Dogs were anesthetized and positioned in sternal recumbency for brain MRI at each participating study institution (Supporting Information: S1). Anesthetic protocols and MRI sequences varied between institutions; however, MRI sequences included a minimum of T2‐weighted sequences in transverse and sagittal orientation, T2‐weighted fluid attenuation inversion recovery, and T1‐weighted pre‐ and postcontrast sequences in transverse orientation following administration of gadopentetate dimeglumine (0.1 mmol/kg IV). Additional diffusion‐weighted sequences with apparent diffusion coefficient mapping sequences in transverse/dorsal orientation were reviewed where available. Cases were excluded if concurrent brain pathology was identified on MRI which could account for the presenting clinical signs, if there was MRI evidence of a hemorrhagic component to the infarcted brain lesion, or if dogs were previously recorded with epileptic seizures.

Collected data included signalment, history, clinical and neurological examination findings, and results of diagnostic investigations. Additionally, MRI findings were recorded, including the location of the ischemic stroke (cerebral hemispheres, thalamus, cerebellum, brainstem, or multifocal), the vascular territory involved, and the extent of the lesion (territorial [infarcts located at the vascular territory of 1 of the main arterial supply to the brain] or lacunar [subcortical infarcts located at the vascular territory of an intraparenchymal deep or superficial perforating artery]).^3^, ^12^ Additional diagnostic information where available was recorded, including thyroid hormone evaluation (total thyroxine/thyroid‐stimulating hormone [T4/TSH]), ACTH stimulation/low‐dose dexamethasone suppression testing (LDDST), noninvasive blood pressure measurements, urine protein and creatinine ratio, cerebrospinal fluid (CSF) analysis, thoracic and/or abdominal imaging, fine needle aspiration/biopsy results from spleen/lymph nodes or identified mass lesions, thromboelastography or viscoelastic coagulation monitoring (TEG/VCM), coagulation profile (fibrinogen, D‐Dimer, and antithrombin II results), and echocardiography. Noninvasive blood pressure was measured based on previously published guidelines following the onset of signs of neurological disease, and consistent measurements of ≥160 mm Hg were considered as evidence of systemic hypertension.^13^ Dogs that were subsequently diagnosed with a new comorbidity following the diagnosis of ischemic stroke, or which had a relevant preexisting comorbidity documented in the clinical history, were recorded.

### 2.2 Follow‐up data collection

Short‐term follow‐up data included survival and time to discharge. Long‐term follow‐up data were collected from medical records and telephone contact with referring veterinarians or owners, and included survival time, development of poststroke epileptic seizures (self‐limiting epileptic seizures within a 24‐hour period), or epilepsy (at least 2 unprovoked epileptic seizures >24 hours apart),^14^ ongoing patient management, and recurrence of ischemic stroke (either confirmed or suspected). Long‐term outcome was defined as: “excellent” if dogs were considered to have made a full recovery, “good” if dogs showed marked neurological improvement but remained with some functional impairment, or “poor” if they did not make a functional recovery or suffered stroke recurrence. Survival time was defined as the number of days between the initial diagnosis of ischemic stroke until the date of death regardless of the underlying cause. For nonsurviving cases, the date and cause of death, alongside postmortem examination results where available were recorded. Dogs were considered to have presumed stroke recurrence if they represented with acute or peracute onset of nonprogressive signs (beyond 24 hours) which were localized to the brain. Dogs that underwent repeat MRI examination and had findings consistent with a second ischemic stroke in a different vascular territory were recorded as a confirmed recurrence.

Owner quality of life (QOL) questionnaires (Supporting Information: S2) were based on previously published owner‐perceived quality of life assessments, and were designed to collect information regarding neurological recovery, social interaction, activity levels, stroke recurrence, and long‐term patient management.^15^, ^16^ In accordance with study ethical guidelines, owner QOL questionnaires and follow‐up information was only collected from owners when the dog was confirmed to be alive by the referring veterinarian at the time of last follow‐up.

### 2.3 Statistical analysis

Statistical analyses were carried out using commercially available software (IBM SPSS Statistics Desktop, Version 28.0, IBM Corporation, Armonk, New York). For categorical data, Chi‐square analysis was used to evaluate the association between comorbidities and ischemic stroke recurrence, lesion location, and epileptic seizures or epilepsy following discharge. Continuous data were tested for normality using the Shapiro‐Wilk test and nonnormally distributed data were represented as median, range, and interquartile range (IQR). Nonparametric tests were subsequently performed, including Mann‐Whitney *U*‐test for 2 independent groups and Kruskal‐Wallis for more than 2 independent groups. Data were tested for an association of short and long‐term survival time, between comorbidities, ischemic stroke location, recurrence, and poststroke epileptic seizures or epilepsy. Kaplan‐Meier product estimates were used to compare survival time between dogs with different underlying comorbidities, MRI findings, and presenting features. Dogs were censored from survival analysis if they were confirmed alive or lost to follow‐up. Statistical analyses were considered significant if *P* < .05.

## 3 RESULTS

### 3.1 Signalment and case presentation

A total of 219 dogs were diagnosed with ischemic stroke between 2000 and 2021 of which 125 dogs were included in the study. Forty‐seven dogs were excluded because of incomplete records, 24 dogs were excluded because of a concomitant relevant lesion found on brain MRI, 18 dogs were excluded because of a hemorrhagic component of the infarcted brain lesion, and 5 dogs were excluded because they had a history of epileptic seizures.

Fifty‐five (44%) dogs were female of which 48 (87%) were neutered, and 70 (56%) were male of which 56 (80%) were neutered. The median age at presentation was 9.6 years (range, 2.1‐15.8 years; IQR 4 years). The median body weight was 17.7 kg (range, 4.2‐55.7 kg; IQR 19.5 kg). The most common presenting breeds were greyhound (14/125, 11.2%), Cross Breed (14/125, 11.2%), cavalier King Charles spaniel (13/125, 10.4%), shih tzu (7/125, 5.6%), English springer spaniel (7/125, 5.6%), German shepherd (6/125, 4.8%), border collie (5/125, 4%), and Labrador retriever (5/125, 4%). Additional breeds represented by less than 5 dogs are included in Supporting Information (S3).

Onset of clinical signs was peracute in 80 dogs (64%), and acute in 45 dogs (36%). The median time between onset of clinical signs and presentation was 1 day (range, 0‐42 days; IQR 1 day). Eleven dogs (11/125, 8.8%) were reported to have epileptic seizures as a primary presenting sign. Thirty‐nine dogs (39/125, 31.2%) were recorded as nonambulatory on presentation. A preexisting comorbidity was documented in 9/125 dogs (7.2%) including undiagnosed cardiac murmur 4/125 (3.2%), myxomatous mitral valve disease 1/125 (0.8%), proteinuria 1/125 (0.8%), chronic kidney disease (CKD) 1/125 (0.8%), hyperadrenocorticism 1/125 (0.8%), and steroid‐responsive meningitis arteritis (well controlled under treatment) 1/125 (0.8%). Neuroanatomical localization included the cerebellum (48/125, 38.4% [11/48 with paradoxical head tilt]), forebrain (35/125, 28%), vestibular system (21/125, 16.8% [19/21 with central involvement, 2/21 with no obvious signs of central involvement]), multifocal (11/125, 8.8%), and brainstem (10/125, 8%). Magnetic resonance imaging findings including the vascular territories involved, and extent of the ischemic infarcts in all dogs are documented in Table 1.

**TABLE 1:** Brain location and arterial territories involved in dogs diagnosed with ischemic stroke.

Brain location, number (%)	Vascular territory involved, number (%)	Extent of ischemic stroke, number (%)
Cerebellum, 67 (53.6%)	Rostral cerebellar artery, 66 (52.8%)	Territorial, 53 (42.4%)
	Caudal cerebellar artery, 1 (0.8%)	Lacunar, 14 (11.2%)
Thalamus, 24 (19.2%)	Caudal perforating artery, 14 (11.2%)	Territorial, 3 (2.4%)
	Proximal perforating artery, 9 (7.2%)	Lacunar, 21 (16.8%)
	Distal perforating artery, 1 (0.8%)	
Cerebral hemispheres, 20 (16%)	Striate artery, 9 (7.2%)	Territorial, 13 (10.4%)
	Middle cerebral artery, 8 (6.4%)	Lacunar, 7 (5.6%)
	Rostral cerebral artery, 3 (2.4%)	
Brainstem, 9 (7.2%)	Caudal cerebellar artery, 4 (3.2%)	Territorial, 0
	Caudal perforating artery, 5 (4%)	Lacunar, 9 (7.2%)
Multifocal, 5 (4%)	Caudal perforating and rostral cerebellar arteries, 3 (2.4%)	Territorial, 3 (2.4%)
	Caudal perforating and striate arteries, 1 (0.8%)	Lacunar, 2 (1.6%)
	Caudal cerebral and rostral cerebellar arteries, 1 (0.8%)	

In the 11 dogs (8.8%) recorded with epileptic seizures as a primary complaint, the infarct was lacunar in 7/11 (63.6%) and territorial in 4/11 dogs (36.4%). Infarct location included cerebral hemispheres (4/11, 36.3%), cerebellum (4/11, 36.3%), thalamus (2/11, 18.2%), and brainstem (1/11, 9.2%).

Additional diagnostic investigations were performed in 91/125 (72.8%) dogs. This included noninvasive blood pressure measurements in 91/125 (72.8%), thoracic and abdominal imaging in 86/125 (68.8%), urine protein and creatinine ratio in 86/125 (68.8%), CSF analysis in 83/125 (66.4%), T4/TSH in 75/125 (60%), TEG/VCM monitoring and coagulation profile in 46/125 (36.8%), ACTH/LDDST testing in 40/125 (32%), and echocardiography in 34/125 (27.2%) dogs. Results of these investigations are included in Supporting Information (S4). Based on diagnostic investigations, newly diagnosed comorbidities were subsequently recorded in 49 dogs (39.2%, Table 2). Cardiac diseases were recorded in 27/125 dogs (21.6%) with myxomatous mitral valve disease (MMVD) being the most common condition (23/27, 85%). Further information regarding cardiac diagnosis and affected breeds are included in Supporting Information (S5). Only 1 dog with cardiac disease had a concurrent arrhythmia (supraventricular tachycardia) which was treated with furosemide (unknown dose). Based on preexisting comorbidities with the additional newly diagnosed comorbidities, a total of 52 (41.6%) dogs were deemed to have a clinically relevant comorbidity.

**TABLE 2:** Comorbidities recorded in dogs with ischemic stroke.

Comorbidities	Number of dogs (%)
Hypertension	25 (20%)
Primary hypertension	16 (12.8%)
Secondary hypertension (chronic kidney disease)	7 (5.6%)
Secondary hypertension (hyperadrenocorticism)	2 (1.6%)
Proteinuria	10 (8%)
Chronic kidney disease	7 (5.6%)
Concurrent confirmed/suspected neoplastic process	7 (5.6%)
Jejunal and hepatic mass, no histopathology available	2 (1.6%)
Urinary bladder transitional cell carcinoma alongside hyperadrenocorticism	1 (0.8%)
Pulmonary carcinoma	1 (0.8%)
Cutaneous lymphoma	1 (0.8%)
Septic peritonitis because of suspected duodenal neoplasia, no histopathology available	1 (0.8%)
Laryngeal mass, no histopathology available	1 (0.8%)
Hypercoagulable state	7 (5.6%)
Hypercoagulable state alone	3 (2.4%)
Alongside aspiration pneumonia	1 (0.8%)
Alongside proteinuria	1 (0.8%)
Alongside hypothyroidism	1 (0.8%)
Alongside panhypoproteinemia because of chronic enteropathy	1 (0.8%)
Hypothyroidism	3 (2.4%)
Hyperadrenocorticism	2 (1.6%)

Median age for dogs with comorbidities (52/125, 41.6%) was 10 years (range, 4.11‐15.8 years; IQR 3.33 years) versus 8.9 years (range, 2.1‐14.2 years; IQR 4.10 years) for dogs without a comorbidity. There were 21 female and 31 male dogs (44 neutered) in the group of dogs with comorbidities. Median body weight for dogs with comorbidities was 19.4 kg (range, 4.2‐55.7 kg; IQR 22.95 kg), versus 17.4 kg (range, 5‐42 kg; IQR 18 kg) for dogs without a comorbidity. There was no significant association between comorbidities and age (*P* = .143), breed (*P* = .201), sex (*P* = .492), or weight (*P* = .566). No association was found between comorbidities, and location (*P* = .128) or extent of the ischemic stroke (*P* = .263). Where indicated, treatment for comorbidities was initiated (Supporting Information: S6).

### 3.2 Follow‐up and outcome

Median hospitalization time for all dogs was 2 days with no significant difference between dogs with (median 2.5 days [range, 1‐9 days; IQR 4 days]) or without (median 2 days [range, 0‐13 days; IQR 3 days]) a comorbidity (*P* = .154). Eight dogs (8/125, 6.4%) did not survive to discharge. One (0.8%) died following cardiopulmonary arrest, 4 (3.2%) were euthanized because of the severity of their comorbidities, 2 (1.6%) were euthanized because of perceived neurological worsening, and 1 (0.8%) was euthanized because of lack of neurological improvement. Four of these dogs were nonambulatory on presentation. The location of the ischemic stroke in the dogs that did not survive to discharge was in the forebrain (4/8, 50%), cerebellum (2/8, 25%), or both (2/8, 25%), and all had a territorial ischemic stroke. Of these dogs, 4 (50%) had a comorbidity. No association was found between survival to discharge and nonambulatory status on presentation (*P* = .255) or a comorbidity (*P* = .116). However, dogs with a cerebellar location of ischemic stroke were significantly more likely to survive to discharge (*P* = .029). Dogs with territorial ischemic stroke were significantly associated with nonsurvival to discharge (*P* = .020). Two of the dogs that did not survive to discharge underwent postmortem examination which confirmed ischemia in the arterial territory of the right middle cerebral artery, corresponding with the MRI findings. Neither of these 2 dogs had embolus or thrombus identified. Postmortem findings of 1 dog are published elsewhere.^17^

**FIGURE 1:**
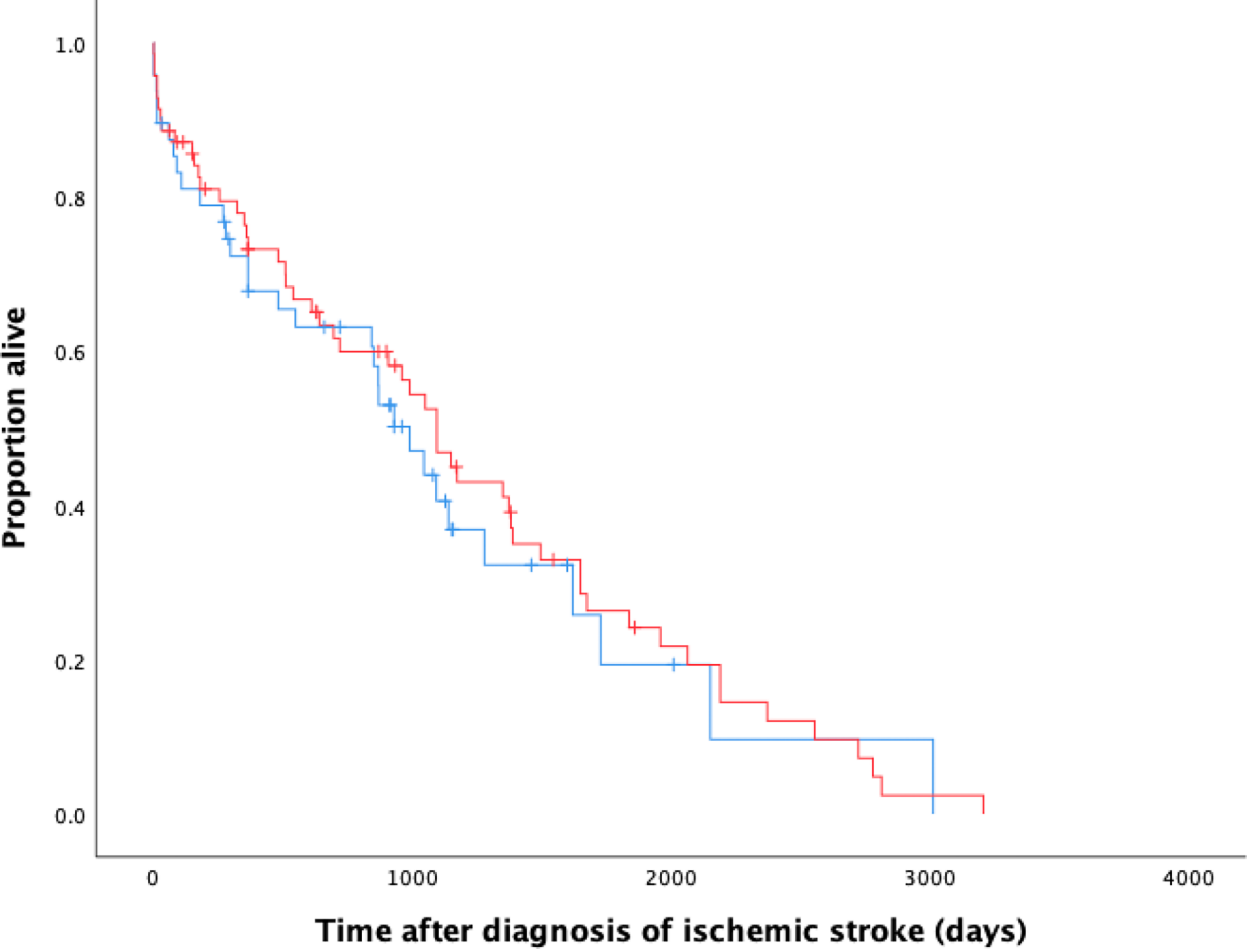
Kaplan‐Meier survival curve in dogs diagnosed with ischemic stroke, with (blue line) or without (red line) a comorbidity. Crosses represent dogs that were lost to follow‐up or were alive at the time of follow‐up, which were censored from analysis.

Regarding long‐term follow‐up, 29 dogs (23.2%) were confirmed alive, 84 dogs (67.2%) were reported dead, and 4 dogs (3.2%) were lost to follow‐up. For dogs known to be alive, the median follow‐up time was 906 days (range, 3‐2010 days; IQR 814 days). The QOL questionnaire was completed for 13/29 (44.8%) cases confirmed alive (Table 3). Overall outcome was considered excellent in 8/13 (61%), good in 2/13 (16%), and poor in 3/13 (23%) dogs. For dogs recorded as deceased, the reason for euthanasia was attributed to suspected (16/84, 19%) or confirmed ischemic stroke recurrence in 2/84 (2.4%). Unrelated reasons for death were recorded in 49/84 dogs (58.3%) and for 17/84 dogs (20.3%) the cause of death was unknown. Median survival time for all dogs regardless of underlying cause, was 708 days (range, 1‐3207 days; IQR 1177 days). Median survival time for dogs with comorbidities was 482 days (range, 1‐3013 days; IQR 900 days) while in dogs without a comorbidity it was 907 days (range, 1‐3027 days; IQR 1395 days). On Kaplan‐Meier survival analysis, no significant difference was found between the survival of dogs with or without a comorbidity (*P* = .602; Figure 1). When evaluated separately with the 2 most common comorbidities (hypertension and proteinuria) no significant difference was found in survival between these groups and the remainder of the dog population (*P* = .725 and *P* = .075). On Kaplan‐Meier survival analysis, no significant difference was found in dogs with or without cardiac diseases (*P* = .295); no significant difference was found between the survival time of dogs with ambulatory or nonambulatory status on presentation (*P* = .064), extent of the ischemic stroke (*P* = .378), or location of the ischemic stroke (*P* = .342; Supporting Information: S7). Of the 92 dogs that were confirmed deceased on follow‐up, 33 (36%) had made a full recovery and the outcome was recorded as excellent, 7 (8%) had a good outcome, and a poor outcome was recorded in 35 dogs (38%, 8/35 died before discharge, 6/35 were euthanized shortly after discharge because of insufficient functional recovery, and 21/35 had suspected or confirmed stroke recurrence). The status of recovery was unknown in 17/92 (18%) dogs.

**TABLE 3:** Owner‐recorded quality of life assessment for dogs diagnosed with ischemic stroke.

Questions from the QOL questionnaire	Number of dogs (%) and answers
Quality of life at present (1 = poor, 5 = excellent)	10 dogs (76.9%): 5/5 1 dog (7.7%): 4/5 1 dog (7.7%): 3/5 1 dog (7.7%): 2/5
Neurological status	8 dogs (61%): returned to normal 5 dogs (39%): improved with some disabilities remaining
Ability to carry out normal activities compared to before suffering an ischemic stroke	10 dogs (76.9%): able to carry out normal activity 3 dogs (23.1%): unable to carry out normal activity
Activity level compared to before suffering an ischemic stroke	2 dogs (15%): increased 9 dogs (70%): remained the same 2 dogs (15%): decreased
Epileptic seizures/epilepsy after discharge	None
Ischemic stroke after discharge	3 dogs (23.1%): had suspected ischemic stroke recurrence 10 dogs (76.9%): no

Abbreviation: QOL, quality of life.

Stroke recurrence was suspected (22 dogs) or confirmed (2 dogs) in 24/125 (19.2%) dogs. The median time for ischemic stroke recurrence was 453 days (range, 14‐2780 days; IQR 877 days). Of the 2 dogs with confirmed recurrence, 1 dog was diagnosed with hyperadrenocorticism, and 1 dog was hypercoagulable based on VCM testing. Of the 22 dogs with suspected recurrence, 9/22 dogs (40.9%) had a comorbidity. There was no significant association found between comorbidities and the likelihood of ischemic stroke recurrence (*P* = .519). When evaluated separately, no significant association was found between ischemic stroke recurrence and the diagnosis of hypertension (*P* = .920) or proteinuria (*P* = .231). Seven dogs (29.2%) had a concurrent cardiac disease, but no significant association was found between the recurrence of ischemic stroke and concurrent cardiac disease (*P* = .863). Median survival time of dogs with stroke recurrence from the initial presentation was 869 days (range, 14‐2814 days; IQR 1200 days) versus 641 days (range, 1‐3206 days; IQR 1216 days) for dogs without recurrence. On Kaplan‐Meier survival analysis there was no significant difference regarding long‐term survival between dogs with or without stroke recurrence (*P* = .812; Supporting Information: S7).

Of the 11 dogs that presented with epileptic seizures as a primary complaint, only 1 suffered further epileptic seizures following hospital discharge and was subsequently classed as epileptic. A total of 7 dogs that did not have epileptic seizures as a presenting feature, developed poststroke epilepsy. The onset of epileptic seizures was at 9 days (1 dog), 30 days (1 dog), 11 months (1 dog), 2.5 years (1 dog) and 4 years (1 dog). In 3 dogs the time of onset of epileptic seizures was unknown. No dogs with poststroke epilepsy had repeat imaging investigations of the brain. Epileptic seizure phenotype was reported as generalized in 6/8 (75%) and focal in 2/8 (25%) dogs. Frequency of epileptic seizures was daily (1 dog), 4 over 2 days (1 dog), once per month (1 dog), twice per month (1 dog), 3 over 2 months (1 dog), once a year (1 dog), multiple within 1 month followed by 21 months seizure‐free before seizure recurrence (1 dog), and for 1 dog the frequency was unknown. Antiseizure medication was administered in 3/8 (37.5%) dogs, including levetiracetam (2 dogs), and a combination of levetiracetam and gabapentin (1 dog). No dosages were available. The epileptic seizure frequency reduced with antiseizure medication, but did not resolve. Location of the ischemic strokes in dogs with poststroke epilepsy was in the cerebral hemispheres (3/8, 37.5%), cerebellum (3/8, 37.5%), and thalamus (2/8, 25%). The extent of the ischemic stroke was territorial in 5/8 (62.5%) and lacunar in 3/8 (37.5%) dogs. No significant association was found between the development of poststroke epilepsy and the location (*P* = .407) or extent (*P* = .772) of the ischemic stroke at initial diagnosis. On Kaplan‐Meier survival analysis there was no significant difference in survival between dogs who had epileptic seizures as a presenting complaint (*P* = .343) and dogs who developed poststroke epilepsy (*P* = .487) compared to those without (Supporting Information: S7). Five of these dogs had a comorbidity (hypertension 2/5, 40%; hyperadrenocorticism 2/5, 40%; proteinuria 1/5, 20%); however, no association was found between comorbidities and development of poststroke epilepsy (*P* = .215).

## 4 DISCUSSION

This study documented the comorbidities, long‐term outcome including suspected stroke recurrence, and development of poststroke epilepsy in dogs diagnosed with ischemic stroke. In the current study cohort, comorbidities were reported in 41% of dogs diagnosed with ischemic stroke, which is similar to previous veterinary literature.^3^, ^4^ The most common comorbidities were hypertension, proteinuria, and cardiac diseases.

In humans, hypertension is a risk factor for the development of ischemic stroke.^18^ The pathophysiological mechanism is considered multifactorial, but the development of atherosclerotic plaques alongside the remodeling of smooth muscles within cerebral arteries might lead to thrombosis and subsequent ischemia.^18^ Similar arterial wall changes leading to ischemia are speculated because of chronic hypertension in dogs^3^; however, the link between hypertension and ischemic stroke in dogs is not well understood. Hypertension is reported in up to 28% of dogs with ischemic stroke; however, 87.5% of these dogs also had concurrent CKD, hyperadrenocorticism, or both.^3^ It is therefore difficult to specifically evaluate hypertension as an independent risk factor for ischemic stroke in dogs. In humans, transient hypertension is documented as a result of ischemic stroke in approximately 75% of patients.^19^ This acute hypertensive response is considered multifactorial, caused either by a possible undiagnosed comorbidity^20^ or as the result of activation of the sympathetic adrenomedullary pathway secondary to the stroke itself.^21^ Although for some cases in our study, it is not known for how long the hypertension persisted before and after diagnosis of ischemic stroke, serial blood pressure measurements are indicated for monitoring and treatment of persistent hypertension which might be differentiated from transient hypertension.

Proteinuria, which was the second most commonly identified comorbidity in our study, is associated with the risk of thrombosis in dogs.^22^ Reduced levels of antithrombin III activity are proposed as a major pathophysiological mechanism leading to thrombosis in dogs. Antithrombin levels lower than the reference range occurs in 12% to 26% of dogs,^22^, ^23^, ^24^ and a hypercoagulable state in up to 89% of dogs with protein‐losing nephropathy.^23^, ^24^, ^25^ In our study, 1 dog with proteinuria was also hypercoagulable based on VCM testing. Based on guidelines for dogs with glomerular disease, antiplatelet therapy is indicated where a comorbidity is associated with the risk of thrombosis.^26^

Thromboembolism is documented in dogs with hyperadrenocorticism and hypercoagulability and is typically linked to values of circulating cortisol level higher in affected dogs.^27^, ^28^ Hyperadrenocorticism was not the most commonly documented comorbidity in our study cohort which is in contrast to previous literature regarding ischemic stroke.^3^ This difference could be attributed to the infrequent testing of adrenal function in the acute setting, or a lack of suggestive history and clinical signs to indicate further testing in the current study cohort.

A substantial proportion of dogs in the current study were diagnosed with cardiac disease, with MMVD being the most common. Despite this, cardiac disease did not significantly reduce long‐term survival. While we are unable to make any conclusions regarding other causes of cardiac disease because of the low numbers of affected dogs, we suggest that MMVD does not appear to be a prognostic factor in dogs diagnosed with ischemic stroke. Despite this, the link between ischemic stroke and MMVD as a possible risk factor is unknown.^4^, ^5^ Cavalier King Charles spaniels were previously overrepresented as a breed diagnosed with ischemic stroke.^3^ This breed is also overrepresented with MMVD.^29^ In cavalier King Charles spaniels, alternative causes have been hypothesized to explain the high incidence of ischemic stroke including Chiari‐like malformation which might alter intracranial blood flow and predispose the breed to cardioembolic infarction.^3^, ^10^, ^30^ Additionally, this breed also has macrothrombocytopenia, caused by a mutation in beta1‐tubulin, which results in an abnormally low concentration of circulating platelets, but with a larger size.^31^ In itself, this breed variation is not associated with clinical signs,^32^ but it has been associated with hypercoagulability measured with TEG.^33^ Therefore, this might be a more important factor for the higher prevalence of ischemic stroke in this breed. Additionally, in another study, cardiac troponin I was investigated in dogs with ischemic stroke.^34^ Despite elevated cardiac troponin I, clinically relevant cardiac diseases were identified only in a small proportion of cases.^34^ Therefore, cardiac diseases were deemed unlikely to be an underlying cause for ischemic stroke in dogs.^34^ In human patients, 1 of the most common cardiac causes of stroke is atrial fibrillation.^35^ Atrial fibrillation is a common complication of dilated cardiomyopathy (DCM) in large breed dogs^36^; however, the incidence of cerebrovascular complications in DCM in dogs is low.^37^, ^38^ The reason for this is unknown, but 1 plausible theory is that dogs have more collateral brain circulation compared to humans.^2^, ^17^ Cardiac thromboembolism was previously reported in dogs with atrial fibrillation, but there are no reports of dogs developing ischemic stroke as a consequence.^38^ In our study, only 1 dog had cardiac arrhythmia. Therefore, cardiac arrhythmias are deemed a rare underlying cause of ischemic stroke.

In our study, a small proportion of dogs did not survive until discharge, being euthanized because of the severity of their signs of neurological disease, or comorbidities. Furthermore, territorial ischemic stroke was associated with a low survival until discharge, which is similar to human literature, where a territorial infarct pattern is related to poor functional outcome at discharge.^39^ In contrast, the cerebellar location of the ischemic stroke was associated with an increased likelihood of survival to discharge. This, however, needs to be interpreted with caution as the cerebellar ischemic stroke was overrepresented in our study. Similarly, cerebellar location of the ischemic stroke in dogs was associated with excellent short‐term prognosis in a previous study.^40^ In human literature, isolated cerebellar stroke has a more favorable short‐term outcome,^41^ because of mild sensory loss and less severe postural impairment compared to other areas of the brain.^42^ Given the high prevalence of ischemic stroke located to the cerebellum in dogs in our and previous studies,^3^, ^4^, ^40^ it is possible that this location will remain an important prognosticating factor to inform clients about the possible outcome in dogs diagnosed with cerebellar ischemic stroke.

In previous literature, long‐term survival in dogs diagnosed with ischemic stroke was significantly shorter in dogs with a comorbidity.^3^ In our study, overall survival time was also shorter in dogs with a comorbidity, but this difference did not reach statistical significance. Different statistical methods were used in previous literature,^3^, ^4^ therefore long‐term survival results might not be entirely comparable. Despite this result, identification of a comorbidity remains important, to ensure timely and adequate treatment to prevent possible secondary complications. Furthermore, it is possible that in our study cohort, survival and outcome of dogs with ischemic stroke which had a comorbidity was not different to those without because the underlying comorbidities were actively recognized and successfully treated thus optimizing case outcome.

Previous studies looking into the recovery of dogs with ischemic stroke reported a poor outcome defined as lack of improvement or recurrence of signs of neurological disease in up to 50% of cases.^3^, ^4^, ^5^ In our study cohort, the proportion of dogs with poor outcome was lower at 38%; however, outcome was not available for the entire cohort of deceased dogs. Thus, the actual number of dogs with a poor outcome might differ. Although the outcome of the dogs that were reported to be alive was considered excellent in most of the cases, the number of owners responding to the QOL questionnaire was small. This might not accurately reflect the overall study cohort and might be inherent to ethical guidelines governing the study, as only owners of the live dogs could be contacted, thus biasing results toward more positive outcomes. Additionally, it is possible that some of the nonresponders did not return to normal function. With this in mind, our results regarding neurological recovery in stroke should be interpreted with caution.

In previous studies, stroke recurrence was documented in 15% of cases and was more likely in those with a comorbidity.^3^ Presumed recurrence of the ischemic stroke was recorded in 19% of dogs in our study cohort, with no significant association between diagnosis of comorbidities. This is in line with more recent literature, where similarly no association was found between stroke recurrence and comorbidities.^5^ Recurrence of ischemic stroke was confirmed in only a minority of cases in the current study. Therefore, we cannot exclude that the cause for the acute onset of recurrent of signs of neurological disease was related to conditions other than ischemic stroke.

In previous veterinary literature, epileptic seizures are not only reported in association with ischemic stroke affecting the cerebral hemispheres,^3^, ^5^ but also in association with ischemic stroke affecting the thalamus.^9^ Isolated epileptic seizures or poststroke epilepsy were infrequently reported in our study cohort. Eleven dogs had isolated epileptic seizures in the acute phase of ischemic stroke; however, only 1 of these dogs went on to develop poststroke epilepsy. Therefore, epileptic seizures in the acute phase of ischemic stroke are not necessarily predictor of poststroke epilepsy. Interestingly, we found no association with the location of ischemic stroke and the development of epileptic seizures, despite epileptic seizures and epilepsy being considered a manifestation of structural or functional disturbance to the forebrain.^43^ Therefore, it is possible that epileptic seizures in some dogs as part of their acute presentation were incorrectly recognized as “epileptic seizures.” This may have occurred as these events were based on the owner description and were witnessed by people unfamiliar with epileptic seizure semiology. Additionally, none of the dogs in the current study had electroencephalography performed to confirm epileptic seizure occurrence. Careful clinical characterization coupled with video recordings, and electroencephalography might aid documentation and occurrence of epileptic seizures in ischemic stroke in dogs.

The occurrence of poststroke epilepsy is 8.7% in humans.^44^ The mechanism for late‐onset epilepsy is characterized by scar formation within the cerebral tissue, reorganization of axonal connections, reduction of inhibitory pathways, and hemosiderin accumulation in the cortical neurons.^45^ Although the exact pathophysiology in canine patients is unknown, a similar prevalence of poststroke epilepsy was reported in the current study. Despite their occurrence, poststroke epileptic seizures did not reduce survival time in our study cohort. Only 3 dogs received antiseizure medications and the rest of the dogs had infrequent epileptic seizures which did not require medical management. This likely contributed to the favorable long‐term outcome and is in line with a recent study, where no association between outcome and presence of epileptic seizures was found in ischemic stroke in dogs.^5^ Guidelines for the treatment of dogs with poststroke epilepsy are lacking, and in the human literature treatment is based on individual patient assessment.^45^

The main limitation of the current study is its retrospective nature. Additionally, the lack of standardized diagnostic protocols including the absence of diffusion‐weighted imaging in some dogs, which may have led to misdiagnosis in certain cases. Although ischemic stroke features on MRI studies are well characterized,^8^, ^10^ up to 12% of ischemic stroke cases can be misdiagnosed as gliomas.^46^ Another limitation was the outcome measures on which the study relied, including ambulation status and subjective neurological improvement, which may be considered crude, subject to recall bias, and insensitive to detect subtle but arguably clinically important changes. Disability scoring systems are becoming available in dogs with noninfectious inflammatory conditions of the central nervous system.^47^ A similar system might be useful for more accurate assessment and outcome measures in dogs with ischemic stroke. Furthermore, follow‐up from veterinarians and owners is inherently susceptible to recall bias which may have introduced an element of error in the recorded data.^48^ The small number of postmortem examinations to confirm the initial diagnosis of ischemic stroke was another limitation. However, this was anticipated given the overall favorable prognosis in most cases.^3^, ^4^, ^5^

In conclusion, dogs diagnosed with ischemic stroke have an overall favorable prognosis. All dogs that did not survive to discharge had a territorial stroke; however, the relevance of this finding is unclear and warrants further investigation. The proportion of dogs diagnosed with a comorbidity is similar to previous studies. While there were no significant differences in survival between dogs with and without a comorbidity, it is possible that the study numbers were too low to detect a difference. Investigations into any possible comorbidities are therefore recommended in dogs with ischemic stroke to ensure timely treatment and reduce the risk of possible complications. Recurrence of ischemic stroke was low and poststroke epilepsy was uncommon. Although cardiac disease might not be a major risk factor for ischemic stroke in dogs, other conditions such as proteinuria and hypertension were frequently documented, and pathophysiologic mechanisms associated with canine ischemic stroke warrant further investigation.

## CONFLICT OF INTEREST DECLARATION

Authors declare no conflict of interest.

## OFF‐LABEL ANTIMICROBIAL DECLARATION

Authors declare no off‐label use of antimicrobials.

## INSTITUTIONAL ANIMAL CARE AND USE COMMITTEE (IACUC) OR OTHER APPROVAL DECLARATION

Approved by the Social Science Research Ethical Review Board of the Royal Veterinary College (SR2022‐0086 and SR2022‐0087).

## HUMAN ETHICS APPROVAL DECLARATION

Authors declare human ethics approval was not needed for this study.

## Supporting information


**Data S1.** Supporting Information.
