# Supplementary material for: Comorbidities, long‐term outcome and poststroke epilepsy associated with ischemic stroke – A multicenter observational study of 125 dogs
Source: J Vet Intern Med. 2024 Dec 23;39(1):e17291. doi: 10.1111/jvim.17291 (PMC11664234; doi:10.1111/jvim.17291)

Supplementary material

**S1.** Magnetic resonance imaging devices:

Philips Intera 1.5 T, Philips Healthcare, Eindhoven, Netherlands

Signa HDe 1.5 T, GE Healthcare, Amersham, United Kingdom

Philips Intera 1.5 T Pulsar System, Philips Medical Systems, Guildford, United Kingdom

Esaote 0.2 T Vet MR, Genoa, Italy

**S2.** Quality of life questionnaire and follow-up for owners

1. How do you perceive your dog's overall quality of life at present (1 - very poor, 5 - excellent)? (*only one selection*)

☐ 1
☐ 2
☐ 3
☐ 4
☐ 5
☐ Prefer not to say
☐ Difficult to say - don't know

1. Following discharge from hospital after the initial diagnosis of a stroke, how do you feel your dog's neurological status has changed if at all? (*only one selection*)

☐ Marked improvement - back to normal

☐ Some improvement - much better but some disability remaining

☐ Little or no improvement

☐ Difficult to say - don't know

1. Is your dog still able to carry out all the normal activities that he/ she did before they suffered a stroke (e.g., walks, toileting with full continence, interaction with family members or other animals)? (*only one selection*)

☐ Yes

☐ No - if no, please state what activities they struggle with or are unable to do

☐ Difficult to say - don't know

1. Compared to the period of time immediately before your dog was diagnosed with a stroke, how do you feel your dog's activity levels have changed if at all? (*only one selection*)

☐ Increased

☐ The same

☐ Decreased

☐ Unable to walk unaided

☐ Difficult to say - don't know

1. Following discharge from hospital after the initial diagnosis of a stroke, has your dog suffered a seizure? (*only one selection*)

☐ Yes - if yes, please provide further details – for example, what was the time period between discharge and seizure occurrence? have there been further seizures and if so, how frequently do they occur?

☐ No

☐ Difficult to say - don't know

N.B. if ‘no’/ ‘difficult to say’, move to question 7

1. Is your dog currently receiving anticonvulsant/ anti-seizure medications? (*only one selection*)

☐ Yes - if yes, can you say please state the name(s) of medication and the dose(s) administered?

☐ No

1. Is your dog currently receiving any treatment following discharge from hospital after the initial diagnosis of a stroke (not including anticonvulsant medication), or ongoing treatment for a previously diagnosed condition? (*only one selection*)

☐ Yes - if yes, please provide further details where possible

☐ No

☐ Difficult to say - don't know

1. Following discharge from hospital after the initial diagnosis of a stroke, has your dog suffered any repeat neurological episodes which have been confirmed or were suspected to be the result of a stroke? (*only one selection*)

☐ Yes - if yes, please provide further details where possible

☐ No

☐ Difficult to say - don't know

1. Would you like to add any additional comments or information regarding your pet which you would like to share. *(open-ended)*

Thank you for your interest and your participation in the study.

**S3.** Breeds of dogs diagnosed with ischemic stroke

| Breeds | Number of dogs (%) |
| --- | --- |
| Cross Breed | 14 (11.2%) |
| Greyhound | 14 (11.2%) |
| Cavalier King Charles Spaniel | 13 (10.4%) |
| Shih Tzu | 7 (5.6%) |
| English Springer Spaniel | 7 (5.6%) |
| German Shepherd | 6 (4.8%) |
| Border Collie | 5 (4%) |
| Labrador Retriever | 5 (4%) |
| Cocker Spaniel | 4 (3.2%) |
| Whippet | 4 (3.2%) |
| Lhasa Apso | 3 (2.4%) |
| Pug | 3 (2.4%) |
| Weimaraner | 3 (2.4%) |
| Jack Russel Terrier | 2 (1.6%) |
| King Charles Spaniel | 2 (1.6%) |
| Rottweiler | 2 (1.6%) |
| Siberian Husky | 2 (1.6%) |
| Staffordshire Bull Terrier | 2 (1.6%) |
| Afghan Hound | 1 (0.8%) |
| American Bulldog | 1 (0.8%) |
| Basset Hound | 1 (0.8%) |
| Bearded Collie | 1 (0.8%) |
| Bedlington Terrier | 1 (0.8%) |
| Boxer | 1 (0.8%) |
| Chihuahua | 1 (0.8%) |
| Dalmatian | 1 (0.8%) |
| Doberman | 1 (0.8%) |
| Dogue de Bordeaux | 1 (0.8%) |
| English Bull Terrier | 1 (0.8%) |
| English Setter | 1 (0.8%) |
| Golden Retriever | 1 (0.8%) |
| Japanese Akita | 1 (0.8%) |
| Labradoodle | 1 (0.8%) |
| Bichon Maltese | 1 (0.8%) |
| Miniature Dachshund | 1 (0.8%) |
| Miniature Schnauzer | 1 (0.8%) |
| Parson Russel Terrier | 1 (0.8%) |
| Patterdale Terrier | 1 (0.8%) |
| Pomeranian | 1 (0.8%) |
| Shetland Sheepdog | 1 (0.8%) |
| Tibetan Terrier | 1 (0.8%) |

**S4.** Results of additional diagnostic investigations in dogs with ischemic stroke

| Diagnostic test | Number of dogs (%) | Results, number of dogs | |
| --- | --- | --- | --- |
| Non-invasive blood pressure measurement | 91 (72.8%) | Normal: 66  Hypertension: 25 (1 dog was recorded as hypertensive prior to referral) | |
| Thoracic and abdominal imaging | 86 (68.6%) | Abdominal ultrasound: 55  Thoracic radiographs: 40  Bi-cavitary CT scan: 28 | Normal: 62  Renal infarcts: 10  Adrenal gland asymmetry: 1  Bilateral adrenomegaly: 1  Splenic infarcts: 2  Splenic nodules: 2  Aspiration pneumonia: 1  Lung nodule: 1  Bladder mass: 1  Jejunal and hepatic mass:1  Extrahepatic portoazygous shunt: 1  Suspected duodenal neoplasia and septic peritonitis:1  Pericardial effusion: 1  Laryngeal mass: 1 |
| Urine protein and creatinine ratio | 86 (68.6%) | Normal: 76  Abnormal: 10 | |
| Cerebrospinal fluid analysis | 83 (66.4%) | Available for review 30/83  Normal: 22  Abnormal: 8 | |
| Total thyroxine/ thyroid-stimulating hormone | 75 (60%) | Normal: 62  Hypothyroid: 3  Euthyroid sick syndrome: 10 | |
| Thromboelastography/viscoelastic coagulation monitoring | 46 (36.8%) | Normal: 39  Hypercoagulable: 7 | |
| Coagulation parameters, fibrinogen, antithrombin, D-dimer, von Willebrand factor | 46 (36.8%) | Prothrombin time and partial thromboplastin time: 7 - normal  Fibrinogen: 2 - normal  Antithrombin: 1 - normal  D-dimer: 2 - normal  Von Willebrand factor: 1 - deficient | |
| Adrenocorticotropic hormone stimulation/ low dose dexamethasone suppression testing | 40 (32%) | Normal: 37  Hyperadrenocorticism: 3 | |
| Other | 15 (12%) | Abdominocentesis: 1 - abnormal  Cardiac troponin: 1 - increased  Angiostrongylus vasorum snap test: 3 - negative  Baermann fecal analysis: 2 - negative  Electromyography of the appendicular musculature: 1 - normal  Fundic examination: 2 - 1 normal, 1 hypertensive retinopathy  Splenic fine needle aspiration: 1 - normal  Shirmer tear test: 1 - normal  Serology (*Anaplasma spp, Borelia burgdorferi, Erlichia spp, Dirofilaria immitis)*: 1 - normal  Tracheal wash and bacterial culture: 1 – multi-drug-resistant E. coli and Staphylococcus aureus  Urine bacterial culture: 2 – urinary tract infection | |

**S5.** Dog breeds recorded with cardiac diseases (ACVIM stage of MMVD was recorded when available, based on Keene BW, Atkins CE, Bonagura JD, et al. ACVIM consensus guidelines for the diagnosis and treatment of myxomatous mitral valve disease in dogs. *J Vet Intern Med* 2019;33(3):1127-1140.)

| Dog breed | Number of dogs | Cardiac condition, number of dogs |
| --- | --- | --- |
| American Bulldog | 1 | MMVD - 1 |
| Cavalier King Charles Spaniel | 4 | MMVD - 2  MMVD Stage B1 - 1  MMVD Stage B2 - 1 |
| Cocker Spaniel | 1 | DCM |
| Cross Breed | 8 | Incidental smoke - 1  MMVD - 5  MMVD Stage B1 - 2 |
| German Shepherd | 1 | Pericardial effusion - 1 |
| Jack Russel Terrier | 1 | MMVD Stage B1 - 1 |
| Labrador Retriever | 1 | MMVD Stage B1 - 1 |
| Lhasa Apso | 1 | MMVD - 1 |
| Miniature Dachshund | 1 | MMVD - 1 |
| Patterdale Terrier | 1 | Aortic stenosis - 1 |
| Pomeranian | 1 | MMVD Stage B1 - 1 |
| Pug | 1 | MMVD - 1 |
| Shih Tzu | 4 | MMVD - 2  MMVD Stage B1 - 2 |
| Springer Spaniel | 1 | MMVD Stage B1 - 1 |

MMVD, myxomatous mitral valve disease; DCM, dilated cardiomyopathy.

**S6.** Treatment for dogs with cerebral ischemic stroke which were recorded with comorbidities

| **Number of dogs** | **Diagnosis** | **Medication** | **Dose** |
| --- | --- | --- | --- |
| 12* | Hypertension | Amlodipine | 0.05-0.1 mg/kg q12-24h oral |
| 10 | Chronic kidney disease | Benazepril | 0.25-0.5 mg/kg q12-24h oral |
| 1 | Chronic kidney disease | Enalapril | 0.5 mg/kg q12h oral |
| 1 | Chronic kidney disease | Telmisartan | 0.5 mg/kg q24h oral |
| 3 | Hypothyroidism | Levothyroxine | 0.02-0.04 mg/kg q24h oral |
| 2 | Hyperadrenocorticism | Trilostane | Unknown dose |
| 1 | Cutaneous lymphoma | Prednisolone | Unknown dose |
| 7 | Hypercoagulable state | Clopidogrel | 1.1-3 mg/kg q24h oral |

*****For remaining dogs initially recorded as hypertensive, no data was available at follow-up regarding medications administered.

**S7**. Figure 2. Kaplan-Meier survival curve in dogs with ischemic stroke. Crosses represent dogs that were lost to follow-up or were alive at the time of follow-up and thus were censored from analysis. (A) Survival of dogs with (red line) or without (blue) hypertension. (B) Survival of dogs with (blue) or without (red) proteinuria. (C) Survival of dogs with (red) or without (blue) cardiac disease. (D) Survival of dogs according to ambulatory (blue) or non-ambulatory status (red) on presentation. (E) Survival of dogs according to the extent of ischemic stroke (lacunar [blue], territorial [red]). (F) Survival of dogs according to the location of ischemic stroke (brainstem [blue], cerebellum [red], thalamus [green], cerebral hemispheres [purple]).  (G) Survival according to recurrence (blue) or no ischemic stroke recurrence (red). (H) Survival according to presence (red) or absence (blue) of seizures as a primary complaint. (I) Survival of dogs with development of post-stroke epilepsy/ epileptic seizures (red) or no epilepsy/ epileptic seizures after discharge (blue).


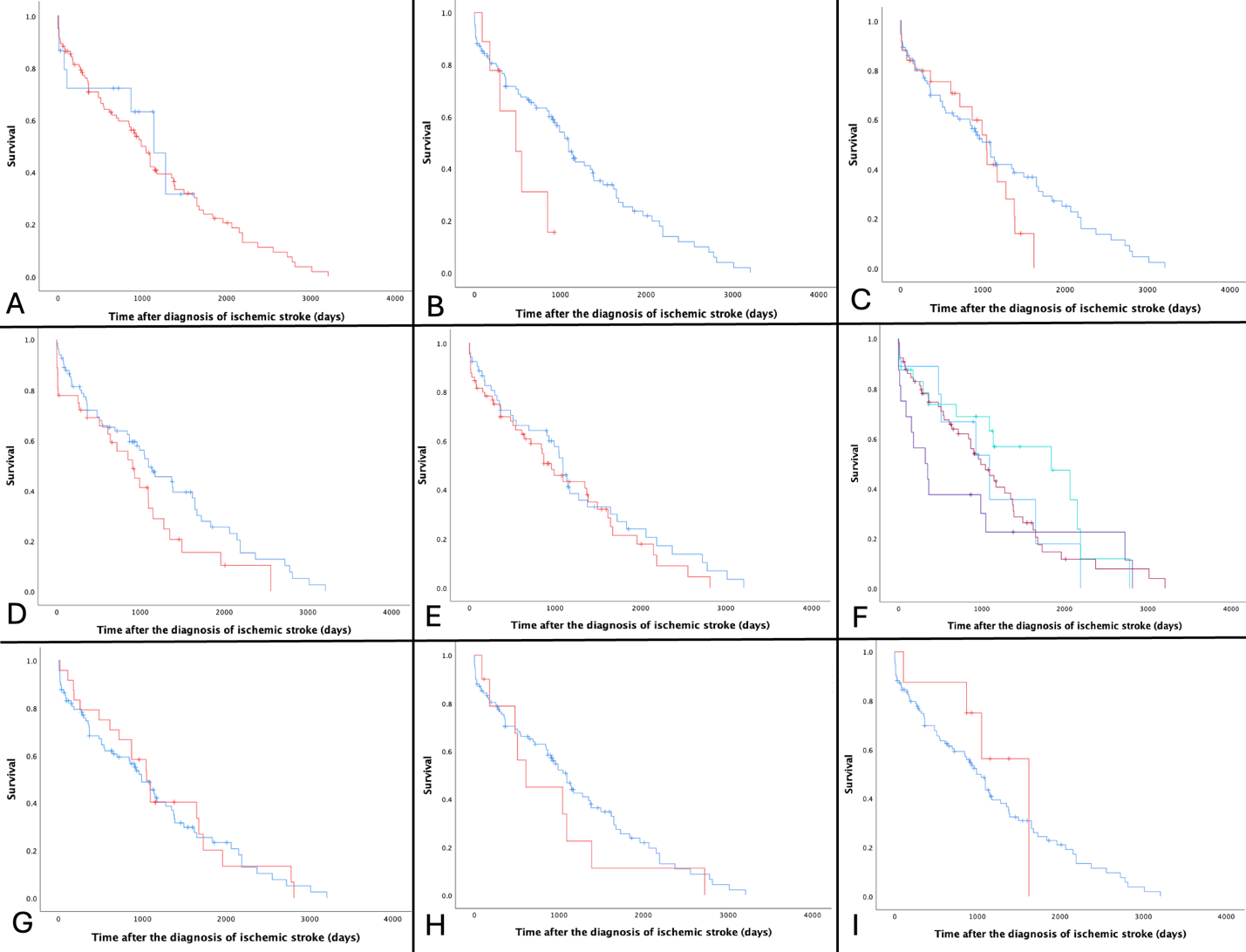

Supplement: Supplementary file 1 — Data S1. Supporting Information. [file JVIM-39-e17291-s001.docx]
